# Fingers Phrase Music Differently: Trial-to-Trial Variability in Piano Scale Playing and Auditory Perception Reveal Motor Chunking

**DOI:** 10.3389/fpsyg.2012.00495

**Published:** 2012-11-16

**Authors:** Floris Tijmen van Vugt, Hans-Christian Jabusch, Eckart Altenmüller

**Affiliations:** ^1^Institute of Music Physiology and Musicians’ Medicine, University of Music, Drama and MediaHanover, Germany; ^2^Lyon Neuroscience Research Center CNRS-UMR 5292, INSERM U1028, University Lyon-1Lyon, France; ^3^Institute of Musicians’ Medicine, Dresden University of Music “Carl Maria von Weber”Dresden, Germany

**Keywords:** piano scale, variability, motor sequence, chunking, perception

## Abstract

We investigated how musical phrasing and motor sequencing interact to yield timing patterns in the conservatory students’ playing piano scales. We propose a novel analysis method that compared the measured note onsets to an objectively regular scale fitted to the data. Subsequently, we segment the timing variability into (i) systematic deviations from objective evenness that are perhaps residuals of expressive timing or of perceptual biases and (ii) non-systematic deviations that can be interpreted as motor execution errors, perhaps due to noise in the nervous system. The former, systematic deviations reveal that the two-octave scales are played as a single musical phrase. The latter, trial-to-trial variabilities reveal that pianists’ timing was less consistent at the boundaries between the octaves, providing evidence that the octave is represented as a single motor sequence. These effects cannot be explained by low-level properties of the motor task such as the thumb passage and also did not show up in simulated scales with temporal jitter. Intriguingly, this instability in motor production around the octave boundary is mirrored by an impairment in the detection of timing deviations at those positions, suggesting that chunks overlap between perception and action. We conclude that the octave boundary instability in the scale playing motor program provides behavioral evidence that our brain chunks musical sequences into octave units that do not coincide with musical phrases. Our results indicate that trial-to-trial variability is a novel and meaningful indicator of this chunking. The procedure can readily be extended to a variety of tasks to help understand how movements are divided into units and what processing occurs at their boundaries.

## Introduction

Playing music means executing particular motor commands to create an auditory stimulus (Jäncke, 2012). Both the motor system and musical materials are highly structured. How can we gain insight into these two kinds of structure: motor sequences and musical phrases? And, more interestingly, how do they interact? Understanding this fundamental problem is crucial to understanding how perception and action are related.

Insight into the organizational structure of motor actions is provided by sequence learning paradigms. Participants learning to type a sequence of numbers divide it into smaller subsequences so as to facilitate learning (Koch and Hoffmann, 2000). Keystrokes at the boundaries of these units or “chunks” (Sternberg et al., 1978; Soechting and Terzuolo, 1987; Hikosaka et al., 1995) are robustly found to be played slower than chunk-internal keystrokes (Povel and Collard, 1982; Rosenbaum et al., 1983; Sakai et al., 2003). Probably, this slowing down is a side-effect of the increased cognitive load of having to transition between the sequence chunks, which in themselves can be executed in an automated, feed-forward fashion (Lashley, 1951).

On the other hand, the musical material is thought to contain structural cues in timing deviations that are, intriguingly, reminiscent of the timing effects found in sequence production literature. That is, pianists slow down at the end of musical phrases (Palmer and Krumhansl, 1987; Repp, 1992a; Furuya et al., 2011). Such phrase-final slowing persist even when instructed to play “metronomically” (Repp, 1999b), and the more advanced a pianist the larger such deviations tend to become (Sloboda, 1983). Tempo variations thus appear to be a communicative device to signal structural landmarks of the musical piece. The process is altogether reminiscent of the widespread phrase-final lengthening in natural language (Klatt, 1976; Turk and Shattuck-Hufnagel, 2007).

Do these results imply that the structure of musical phrases is that of the motor commands used to create it? In other words, could musical phrases and motor sequences be two sides of the same coin? This idea conflicts with the *perceptual hypothesis* (Drake, 1993), which explains temporal deviations in music production as resulting from perceptual biases. For example, suppose that the last interval in an isochronous sequence always sounds too short. In other words, our perceptual timing space is warped (Penel and Drake, 1998). As a compensatory strategy, pianists, aiming for perceptual regularity, rather than “objective” regularity, will gravitate toward lengthening that final interval (Repp, 1999b). In brief, biases in perception likely cause training to favor certain irregular patterns rather than objective evenness.

Then what causes temporal deviations in musical playing: motor chunking or perceptual deviations? We propose musical scale playing as our paradigm to investigate this question. Scales are the quintessential musical practice materials: the first thing Mozart’s Zauberflöte character Tamino plays on his flute is a scale. Indeed, practising scales is one of the chores that every classical pianist is engaged in for many hours during their professional career. As a result, we can expect that their motor structure has become sufficiently stable. In order to disentangle motor sequence structure and musical phrasing, we present a novel analysis of scale timing. This method segments the unevenness of playing into:

(i) systematic deviations from objective evenness that are perhaps *residuals of expressive* timing (Repp, 1999a) or resulting from perceptual biases (Penel and Drake, 1998), which we refer to as irregularity, and
(ii) non-systematic deviations that can be interpreted as motor execution errors, perhaps due to noise in the nervous system (Harris and Wolpert, 1998; Faisal et al., 2008), which we will call *instability*.

The analysis is described in more detail below.

In this study we will first of all present a validation of our irregularity-instability analysis by showing that it allows one to reconstruct the previously used unevenness measure (Experiment I). We hypothesize that instability of the various notes in a scale will indicate how the motor program is chunked, whereas irregularity reveals its musical structure (Experiment I and II). Finally, in order to gain insight into the link between perception and action, and we investigate the relation between auditory perception resolution on playing instability (Experiment III).

Furthermore, our study is the first to investigate various types of scales, thus being able to control for differences in motor program (i.e., the fingering) and musical content (for example, major vs. minor scales). In addition to the C-major scale, we will include two other scales as controls. The first is the A-minor scale, which is of interest to us since it is played with a fingering identical to C-major. That means, in terms of low-level motor execution it is exactly the same as C-major (except of course for its being played three tones lower than C-major) whereas in terms of the musical content and the tension-resolution profile it is very different (Krumhansl and Kessler, 1982). As a counterpart to this control we took the F#-major scale, which is very different as regards the finger movements, but the relative tone distances between the notes are the same as C-major.

## Experiment I

### Materials and methods

#### Experimental set-up

Thirty-four right-handed pianists were recruited from the student pool at the Hanover University of Music. Participants (17 female) were on average 24.72 (SD 4.47) years old. They started piano training at 6.4 (SD 2.2) years of age, and accumulated an average of 14140 (SD 8894) practice hours at their instrument. None of these participants reported any neurological disorder or problems related to performing, such as chronic pain. Participants played a Kawaii MP9000 stage piano connected to a Pioneer A109 amplifier. The MIDI data was captured through an M-Audio MIDI to USB converter and fed in to a Linux-PC running a custom developed C program that captured the MIDI events. The participants were invited to first play a few minutes to get used to the set-up and warm up. Then they played the scale exercises, which are explained in detail below. The exercises were presented in note score format with indicated (standard) fingering. The pianists were asked to play as regularly as possible at a comfortable mezzo-forte loudness and in legato style. The entire procedure took about half an hour, and the pianists received a nominal financial compensation (10 Euro). The experiment was performed in accordance with the Declaration of Helsinki.

#### Design

Participants played two-octave piano scales accompanied by a metronome at 120 BPM. They played four notes within a metronome beat, i.e., eight keystrokes per second. They played blocks of approximately 30 alternating ascending and descending scales with a 9-note rest in between. The scales were played in the following blocks, separated by small breaks: (i) C-major with the right hand, (ii) C-major with the left-hand, (iii) A-minor with the right hand, (iv) F#-major with the right hand, (v) C-major with both hands. The left-hand and both-hand conditions were included as part of our scale playing battery, but will not be reported on in this paper. The C-major and A-minor scales were played with their conventional fingering (123123412312345, where the numbers indicate the fingers from the thumb, 1, to the little finger, 5, and the F#-major with 234123123412312). Following musicological convention, we will refer to the notes by their rank in the scale, in ascending order: $\hat{1},\hat{2},\hat{3},\hat{4},\hat{5},\hat{6},\hat{7},{\hat{1}}^{\prime },{\hat{2}}^{\prime },{\hat{3}}^{\prime },{\hat{4}}^{\prime },{\hat{5}}^{\prime },{\hat{6}}^{\prime },{\hat{7}}^{\prime },{\hat{1}}^{\prime \prime }.$ The C-major scales started at middle c at 262 Hz (c4). The f-minor scale started at 220 Hz (a3) and the F#-major at 370 Hz (F#4).

#### Establishing scale playing unevenness

Previous scale playing studies computed the SD of the intervals between the subsequent onsets of the keystrokes as a measure of playing unevenness (Seashore, 1938; Wagner, 1971; MacKenzie and Van Eerd, 1990). More recently, this unevenness metric has been shown to be an indicator for pianistic expertise (Jabusch et al., 2009) as well as for sensorimotor coordination deficits in pianists (Jabusch et al., 2004).

However, a shortcoming of this metric is that it cannot be applied to investigate single-note timing deviations relative to an established temporal reference. For example, suppose one note in the scale is played too late, which is referred to as an “event onset shift” (Repp, 2005). As a result, two intervals are influenced: the interval before this note would come out too long, but the interval following the note would be too short. In brief, a single deviation shows up in two places in an interval-based deviation trace, making it impossible to disentangle the timing of individual notes. Our analysis remedies this problem as described in detail below.

#### Scale analysis

First we will describe our analysis of a single played scale. Suppose we have isolated the keystrokes and onsets of one correctly played scale. We then convert the note values to their rank in the scale (so for a C-major scale c would have rank 0, d has rank 1, e has rank 2, etc., up to c″ with rank 14) and perform a least-square straight line fit to this set of pairs of rank and timing. This allows us to compute for each note the expected time according to this fit and then the deviation of the timing of the actually measured onset (in ms).

Now we turn to our procedure to analyze the entire MIDI recording for a single participant. First we identified correctly played ascending and descending scales. We then performed the analysis described above for each scale separately and group the obtained temporal deviation values by playing direction (ascending or descending) and by note, yielding a set of 30 such deviations, one for each repetition.

### Results

#### Irregularity and instability example

**Figure 1:**
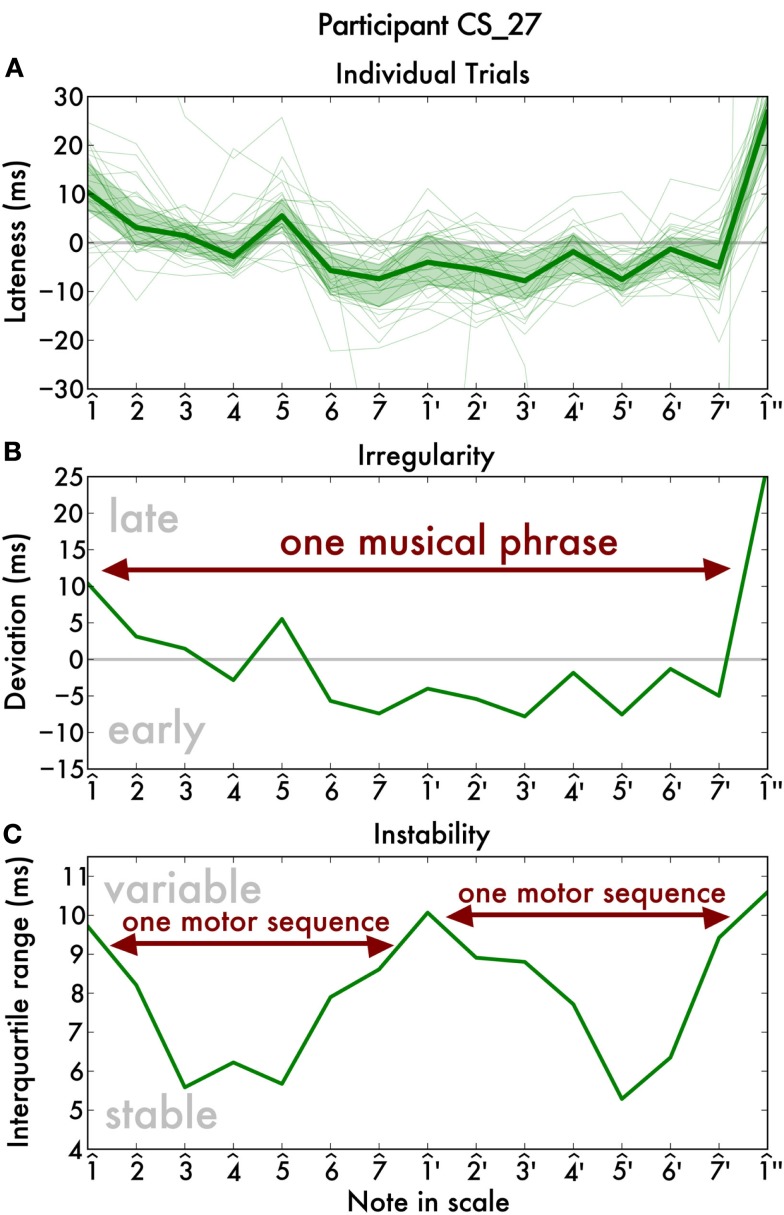
**Overview of our analysis of one pianists’ scale playing reveals that a single musical phrase is divided into at least two motor sequences**. **(A)** For each note, the deviation (in ms) from the straight line that we fit to the key onsets. The thin lines show the deviation profile of one single trial. The thick line shows their median and the shaded area indicates the interquartile range (a measure of variability). These two quantities are then separated to yield two separate traces per pianist: an irregularity trace **(B)** that shows the average lateness, and an instability trace **(C)** that shows the average deviation from his or her playing, i.e., the width of the error area in the top plot.

As an illustration of how irregularity and instability were computed, we will present the data of a single participant playing a two-octave C-major scale. Each line in the Figure 1A represents a single trial and shows the deviation of each note from the fitted straight line (in ms). By combining the 30 repetitions, we found that some notes are systematically late, such as for example, note $\hat{5}$ (g) is reliably around 8 ms late. We call such systematic deviation *irregularity*. Independently of this, some notes show high trial-to-trial variability, such as for example ${\hat{1}}^{\prime }$ (c′), whereas other notes show low variability, i.e., they are played very consistently. To quantify this, we calculate the interquartile range of the note timings and refer to this as *instability*. That is, at each note position, irregularity was quantified as the mean timing deviation, and instability as the interquartile range, across all 30 trials.

Irregularity corresponds to what most previous studies have investigated. We found the irregularity trace is roughly an arc (Figure 1B), in agreement with widespread findings in the timing literature (Palmer and Krumhansl, 1987; MacKenzie and Van Eerd, 1990; Friberg et al., 2006). That is, playing is slower in the beginning, then speeds up and finally slows down at the end. We conclude from this that the two-octave scale is played as a single musical phrase. Surprisingly, the instability trace reveals a different picture, showing three distinct peaks that coincide with the octave boundaries (Figure 1C), instead of two peaks in the irregularity trace.

In sum, we show that, although the two-octave scale is played as a single musical phrase, it is divided into two motor sequences with higher instability at their transition. Our study is the first that we are aware of to reveal this separation.

#### Extraction of correctly played scales

One participant was eliminated because he did not follow the instructions to play in a legato style. Scales that were incorrectly played were rejected (3.9% of the note onset events) as were scales for which the least-square fit had an *R*^2^ less than 0.9 (0.07%), which reflected anomalies in playing. One participant’s F#-major scales were rejected altogether because he consistently played b# instead of b. Data from the correctly played scales was used to calculate the irregularity and inconsistency for each pianist, exercise, and note separately. The least-square fit to the scales had an average *R*^2^ of 0.9996. The procedure yielded an average of 31.2(±3.09) correctly played ascending and descending scales per exercise per participant.

#### Comparison with standard deviation of inter-onset intervals

First, we compare our irregularity-instability analysis to the existing measure of unevenness. For each participant, scale, hand, and playing direction (ascending, descending), we computed unevenness, irregularity, and instability as follows. The unevenness was calculated by taking the SD of the inter-keystroke-intervals in each scale run and then averaged for all runs in each playing direction. Irregularity was computed as above for each note in the scale and then averaged across runs in each playing direction. Instability was calculated as the interquartile range of the deviations of each note, and then averaged across the notes in each scale and playing direction. That is, for each participant we obtained six scale conditions (the five scale tasks listed in the methods, one of which was played bimanually) times two playing directions (ascending, descending), that is 12 data points, for each of which we had three scalars: the unevenness, irregularity, and instability. We then proceeded with a multiple linear regression to predict the former on the basis of the latter two. Both irregularity and instability resulted as significant factors (both *t* > 16.0, *p* < 0.001). The adjusted *R*^2^ of the obtained model is 0.81. Taking either of the two factors alone yields worse *R*^2^ of 0.69 and 0.61, respectively. The two-factor model is significantly better than the single-factor models with irregularity [*F*(1,32) = 254.56, *p* < 0.001] or instability [*F*(1,32) = 416.64, *p* < 0.001] only. Our model is summarized in the equation IOI-SD = 0.93*irregularity + 0.64*instability + 0.62. In brief, using our irregularity-instability analysis, we can reconstruct the unevenness measure with high precision.

#### Instability across notes

**Figure 2:**
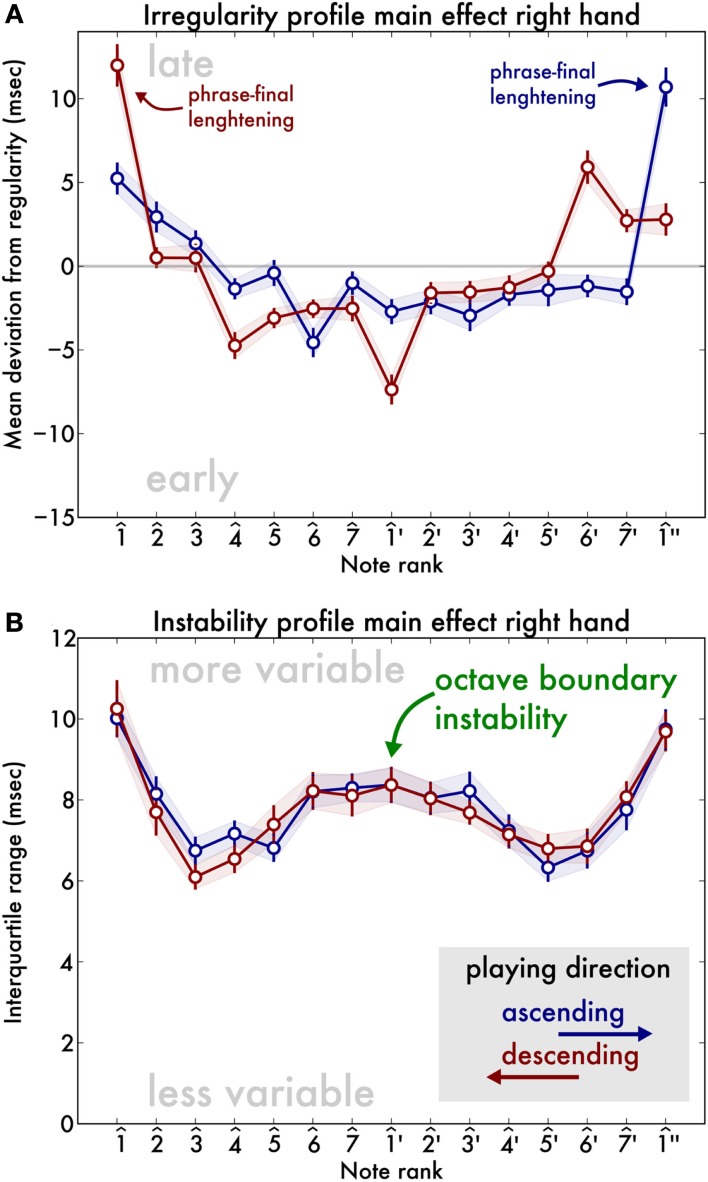
**Irregularity (A) and instability (B) of playing as a function of note position in scale, collapsing the results from all three scales**. Error bars and shaded area indicate standard error of mean. Crucially, the irregularity takes a u-shape whereas the instability shows a prominent peak at the octave boundary, revealing a w-shape.

First, we performed an overall three scales (C-major, A-minor and F#-major) × 2 directions (ascending, descending) × 15 notes ANOVA with irregularity as an outcome variable, and the participants as error terms. We found no main effect of scale [*F*(2,62) = 1.46, *p* = 0.24], nor of direction [*F*(1,31) = 0.12, *p* = 0.73]. There was, however, a main effect of note position [*F*(14,434) = 42.50, *p* < 0.001], indicating that note timing varied across the scale. Furthermore, an interaction of scale with note [*F*(28,868) = 6.10, *p* < 0.001] indicated that this timing trace differs across the three scales under investigation. There was no interaction between scale and direction, but there was between direction and note [*F*(14,434) = 12.27, *p* < 0.001] as well as a three-way interaction between scale, note, and direction [*F*(28,868) = 6.03, *p* < 0.001]. Figure 2A shows the main effect of note position for the two directions separately, collapsed across the three scales, revealing a u-shaped curve as in our example Figure 1B.

Second, we performed the same ANOVA (3 scales × 2 directions × 15 notes) but with instability as an outcome measure. We found no main effects of scale or direction, but again a main effect of note [*F*(14,434) = 48.21, *p* < 0.001], indicating that note timing variability was different at different positions in the scale. Of the two-way interactions, only scale with note was significant [*F*(28,868) = 2.32, *p* < 0.001] and the three-way interaction scale, note, and direction was significant [*F*(28,868) = 2.14, *p* < 0.001]. Figure 2A shows the main effect of note position on irregularity for the two directions separately but collapsed across the three scales. Instability, however, showed a qualitatively different, w-shaped trace (Figure 2B).

We used trend analysis using orthogonal polynomials to investigate the contributions of the various polynomial degrees to the main effect of note on instability. We found no linear or cubic effect but a quadratic (u-shaped) and quartic (w-shaped) effect [*F*(1,31) = 110.24, *p* < 0.001 and *F*(1,31) = 511.87, *p* < 0.001, respectively] as well as significant fifth- and eighth-degree contributions [*F*(1,31) = 4.3, *p* = 0.04 and *F*(1,31) = 8.56, *p* < 0.001]. The dominant trend is by far the quartic trend, corresponding to 78.92% of the total sum of squares of the note effect. Indeed, *post hoc* paired *t*-tests on the C-major scale reveal greater instability at the beginning of the two-octave scale [instability at $\hat{1}$ is greater than at $\hat{4},$ *t*(32) = 9.5, *p* < 0.001] as well as at the end [instability at ${\hat{1}}^{\prime \prime }$ is greater than at ${\hat{5}}^{\prime }$, *t*(32) = 7.3, *p* < 0.001]. Surprisingly, a third peak of instability was found at the transition between the two-octaves [instability at ${\hat{1}}^{\prime }$ is greater than at $\hat{4},$ *t*(32) = 6.1, *p* < 0.001 and also than at ${\hat{5}}^{\prime },$ *t*(32) = 6.3, *p* < 0.001]. Instability was the same at the beginning and end of the scale [instability at $\hat{1}$ and ${\hat{1}}^{\prime \prime }$ are not significantly different, *t*(32) = −0.8 *p* > 0.4].

At this point, one may wonder whether there are systematic timing differences at the octave boundary. That is, does the peak in instability at the middle octave boundary also appear in the irregularity trace? Trend analysis using orthogonal polynomials in the irregularity trace, revealed a strong quadratic trend [*F*(1,31) = 899.24, *p* < 0.001], in line with visual observation of the u-shape of Figure 2A, as well as a cubic trend [*F*(1,31) = 27.37, *p* < 0.001]. However, a quartic (w-shaped) trend was found as well [*F*(1,31) = 49.79, *p* < 0.001], as well as various higher order polynomials. In this case, the dominant trend is the quadratic trend, containing 82.18% of the variance, whereas the quartic trend only amounts to 4.55%. Indeed, using *post hoc* *t*-tests, we find irregularity it is not greater at ${\hat{1}}^{\prime }$ than at $\hat{4}$ [*t*(32) = −0.06, *p* > 0.5] or at ${\hat{5}}^{\prime }$ [*t*(32) = −7.1, *p* = 1]. However, a strong deviation from regularity occurs at the end of the scale, where the deviation at ${\hat{1}}^{\prime \prime }$ (9.3 ms) is greater than at ${\hat{5}}^{\prime }$ (1.1 ms) for ascending scales [*t*(32) = 3.3, *p* < 0.005]. In other words, ${\hat{1}}^{\prime \prime }$ is played systematically late. Similarly, in descending scales irregularity at $\hat{1}$ is greater than at $\hat{4}$ [*t*(32) = 9.4, *p* < 0.001]. This is a common finding in the timing literature: musicians slow down at the end of musical phrases (e.g., Repp, 1999a).

**Figure 3:**
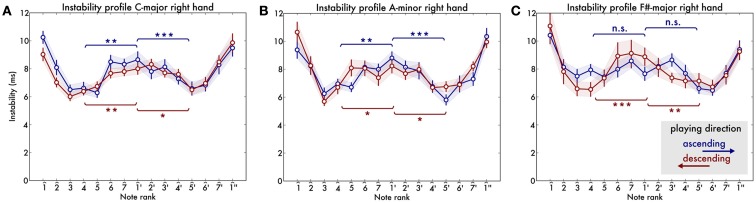
**Instability profile for the three scales individually: (A) C-major, (B) A-minor, and (C) F#-major scales**. Asterisks indicate significance levels of *post hoc* t-tests after Bonferroni correction is applied.

Now we turn to the A-minor scale (Figure 3B). In both ascending and descending scales we found the octave boundary instability peak [instability at ${\hat{1}}^{\prime }$ is greater than at $\hat{4}$, both *t*s(32) > 3.1, *p*s < 0.001 and also comparing to ${\hat{5}}^{\prime },$ both *t*s(32) > 3.1, *p*s < 0.003] (Figure 3C). We can conclude that the octave boundary instability is also present in the A-minor scale.

In the F#-major scale we found again the instability peak at the octave boundary in the descending scales [*t*s(32) > 3.9, *ps* < 0.001] but not for the ascending scales [*t*(31) = -0.06, *p* = 0.73 and *t*(31) = 2.54, *p* = 0.1 for the second octave].

### Discussion

First of all, we have validated our analysis method. We have decomposed the variability from a single variable (unevenness) into two mostly independent and qualitatively different factors (irregularity and instability). This is comparable to the way a vector in the Euclidian plane can be written as a linear combination of two basis vectors.

The participants’ trial-to-trial variability profiles (instability) show a clear w-shape pattern across the two scales, with greater instability at the beginning and end of the scale, but surprisingly also in the middle, at the boundary between the octaves. To our knowledge, our study is the first to reveal such subtle but robust differences in timing consistency. Of course, the irregularity and instability curves are related: when the mean deviation is high, the variance typically also increases. This could explain how instability peaks at the beginning and end of the two-octave scale are accompanied by irregularity peaks at those locations. This means that our finding of the instability peak at the octave boundary is all the more striking since the u-shaped irregularity curve is at its low-point there.

One may argue these two peaks could alternatively be explained by the mechanical effect of inverting the wrist movement, which switches at those locations between left-to-right and right-to-left movement account, first of all, this movement direction inversion is not abrupt, since ascending and descending scales in our measurement are separated by a 9-note rest (1.125 s). Secondly, such an explanation could not explain why a comparable peak occurs in the middle of the two-octaves.

One other potential explanation for the w-shaped variability pattern is that at the boundary between the two-octaves another event occurs: the thumb passes underneath the fourth finger to be able to play the ${\hat{1}}^{\prime }$ key (in the C-major and A-minor scales), for the inverse playing direction a cross-over maneuver of the fourth finger over the thumb is required. Perhaps these are particularly difficult movements to perform, which would explain the higher variability. For example, a thumb-under movement is accompanied by a substantial horizontal wrist translation that requires preparation (Engel et al., 1997) and a coordinated effort across the different fingers (Furuya et al., 2011). However, we argue these phenomena cannot explain our data sufficiently, because a similar movement occurs in the first octave when also the thumb passes underneath the middle finger (at $\hat{3}$) to play $\hat{4}.$ And we do not observe increased instability at this note relative to surrounding notes. Furthermore, the very last note, ${\hat{1}}^{\prime \prime },$ is played without a thumb passage at all and still shows a striking increase in instability. Finally, the F#-major scale contains thumb passages that are much less awkward and in different positions than the C-major scale. Therefore, the w-shape pattern in the descending F#-major scales is not explicable on the basis of thumb passages. We feel these arguments rule out low-level motor explanations of our results.

Another potential explanation for the w-shaped instability would be that this pattern is related to the metronome. However, note that a metronome click occurs every four notes, that is, at $\hat{1}$, $\hat{5}$, ${\hat{2}}^{\prime }$ and ${\hat{6}}^{\prime }$ in the C-major scale. These do not coincide with the w-shape instability, making this explanation untenable.

Then the question arises how the increased instability at the octave boundary is to be explained. If the motor system would conceive of the two-octaves as a single motor program there would be no reason for the playing to become increasingly variable in the middle. Rather, our interpretation is that the octave boundary marks the transition between concatenated motor programs. Under this view, the increased instability is a result of having to load the next sequence into the motor buffer (Lashley, 1951). In other words, the two-octaves are “chunked” into at least two units in the motor system. Again, although we argue that our results indicate segmentation into at least two motor programs, we cannot conclude that the two-octaves make up only two motor programs. It is conceivable that the two-octaves are further subdivided in ways that are not reflected in playing instability, perhaps in a hierarchical fashion (Rosenbaum et al., 1983; Hard et al., 2011).

It is interesting to note that the scales under investigation have revealed the octave boundary effect for both ascending and descending scales (except in the F#-major scales), suggesting that the motor system has chunked these in the same way. Ascending and descending scales are essentially the same movement, but mirrored in time. Therefore, our finding suggests that motor program chunking would be mostly invariant to temporal inversion.

At this point it remains possible, at least in principle, that this instability effect is an artifact of our line fitting procedure or another aspect of our analysis. To control for this, we run the same procedure with simulated data in Experiment II.

## Experiment II

### Materials and methods

We used a python script to simulate the scale playing of 33 pianists. Each simulated pianist played 30 ascending and descending two-octave C-major scales at 8 notes/s, yielding a total of 900 note onsets that were perfectly regular in time. The timing of each note was then jittered by a time value sampled from a normal distribution with zero mean and a SD of 9 ms. This value was chosen such that the resulting instability profile was on average similar to that found in the real pianists. The same analysis as described for Experiment I was then applied to these data.

### Results

**Figure 4:**
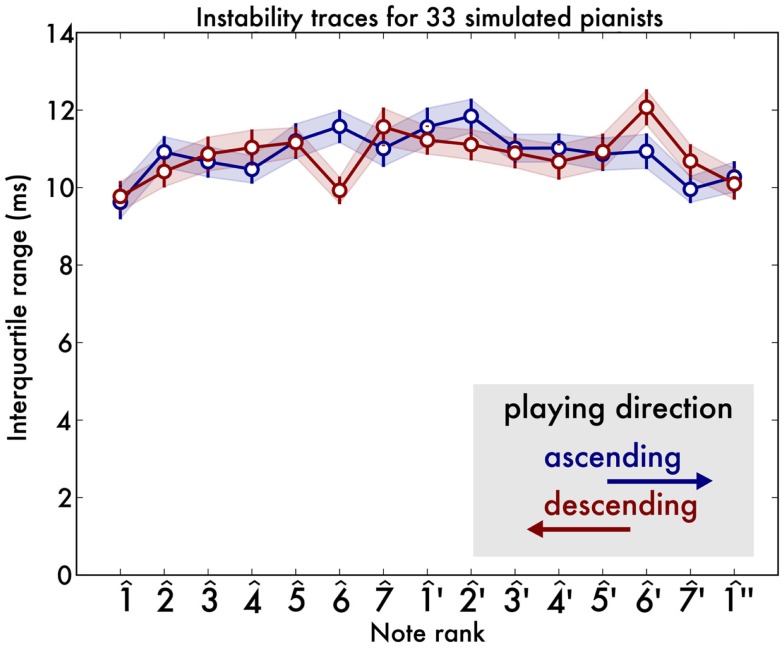
**Instability of 33 computer-simulated scales with randomly jittered timing**. The shaded area indicates the standard error of the mean. The scales show that the variability is distributed uniformly across the scale and there is no evidence of a u-shape or w-shape.

Overall instability levels were comparable to those of the human pianists reported in Experiment I and are shown in Figure 4. Although statistics are not commonly computed for simulated data, we reproduce the same analyses as with the human data in order to understand which effects could be due to random variability. We performed a 2 directions × 15 notes within-participants ANOVA with irregularity as the outcome variability. None of the main effects or interactions were significant (all *F* < 1.5). Performing a similar analysis on instability, we find a main effect of note [*F*(14,448) = 3.26, *p* < 0.001]. Trend analysis revealed this effect to be mostly due to a quadratic trend [*F*(1,32) = 27.30, *p* < 0.001], but, crucially, the quartic trend was not significant [*F*(1,32) = 1.81, *p* = 0.18]. This is further confirmed by our *post hoc* *t*-tests [${\hat{1}}^{\prime }$ is not greater than $\hat{4}$, *t*(32) = 1.6, *p* > 0.05, similarly ${\hat{1}}^{\prime }$ is not greater than ${\hat{5}}^{\prime },$ *t*(32) = 1.0, *p* > 0.15].

### Discussion

We ruled out the possibility that the octave boundary instability that was seen in the recordings of pianists would be an artifact to our analysis. If so, it would also have occurred in the simulated corpus.

Our novel analysis of scale playing has divided the playing unevenness into largely independent components: systematic deviations from regularity and trial-to-trial instability. *A priori*, one would expect that only the former, systematic deviations could be the result of a compensation mechanism that plays longer temporal intervals that sound shorter and vice versa (Drake, 1993). The reason is that such perceptual biases should be the same between trials and therefore give rise to consistent temporal deviations. Therefore, the instability peaks cannot be the result of perceptual warping. In other words, we expect the detection profile to correlate with the produced irregularities and not the instabilities.

In order to test this hypothesis we performed a temporary delay detection experiment in which one note was delayed at various positions in the scale, and participants were asked to detect this. Since Experiment I revealed the octave boundary to be present in the three scales (although to a lesser extent in F#-major), we decided to restrict our current investigation to the C-major scale only.

## Experiment III

### Materials and methods

Nineteen music students of various instruments were recruited from the Hanover University of Music. We used a python script to generate two-octave C-major scales (from $\hat{1}$ up to and including ${\hat{1}}^{\prime \prime }$), ascending only. The tones were played perfectly regularly at 8 notes/s except for one of five notes $\left(\hat{1},\hat{4},{\hat{1}}^{\prime },{\hat{4}}^{\prime },\text{or}\;{\hat{1}}^{\prime \prime }\right)$ that was delayed by 40 ms. This procedure is similar to previous perceptual studies (Ehrlé and Samson, 2005). Five additional two-octave C-major scales with no deviation were inserted to yield a total of 10 stimuli and the entire set was presented twice in random order. The scales were preceded by two short high-pitch piano tones with a 500 ms interval to establish the temporal reference.

The python script generated MIDI files (using the MXM Python MIDI package), which were then converted offline into wave using Timidity and presented using Audacious. Participants indicated on a paper form for each scale whether they heard a timing deviation. As a training, they first heard two example scales with a (longer) deviation and two scales without and received accuracy feedback.

### Results

Overall, the participants responded 79% (SD 8.2%) correctly, showing the feasibility of the task. For each of the five delay locations we calculated the hit rate (correct answers/number of presentations). It is not possible to calculate a *d*′-score since the presentation of the five delay locations were randomly interleaved.

**Figure 5:**
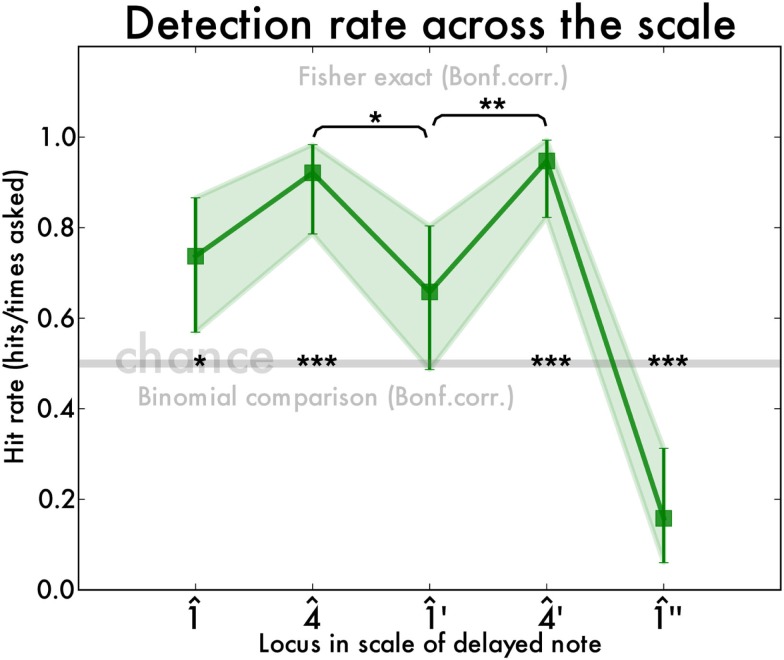
**Hit rate (number of correct answers divided by number of presentations) for detection of timing perturbations at different notes in the two-octave C-major scale**. Chance level is at 0.5 and is indicated by a gray line. The shaded area and the error bars demarcate the 95% binomial confidence interval.

Detection rates were above chance level at all locations in the scale (binomial test all *p*s < 0.005) except for, crucially, at the middle octave boundary at ${\hat{1}}^{\prime }$ (binomial *p* > 0.07). Adjacent $\hat{4}$ and ${\hat{4}}^{\prime }$ were significantly better (Fisher exact *p* = 0.01 and *p* = 0.003 respectively). Interestingly, detection rate at the end of the scale $\left({\hat{1}}^{\prime \prime }\right)$ was worse than chance, meaning that participants were biased to not perceive a perturbation there even if there was (Figure 5).

### Discussion

This experiment reveals that detection accuracy varies by note position in the scale. Overall accuracy was good, showing that the task was feasible. Optimal performance was seen in the middle of either of the octaves (at $\hat{4}$ and ${\hat{4}}^{\prime }$). Detection was at chance level at the beginning of the two-octave scale (at $\hat{1}$) although the participants had a pre-established temporal reference because of the metronome clicks. Detection was also impaired at the end of the two-octave scale, which is a result that ties in well with previous accounts of how perception accuracy is influenced by expectation (Repp, 1999b): listeners expect a slowing down at the end and therefore when there is a delay in that position the stimulus is not perceived as deviant. This is reflected by the fact that performance was below chance level. If participants responded randomly they would have a detection rate of 50%, but in our case they actually exhibit a bias toward not hearing the deviation, even if there is one.

The crucial case, however, is the transition point between the two-octaves. We observed a decreased auditory sensitivity to delays this point, but pianists’ playing shows no systematic deviation (Experiment I). Thus, one must reject our initial hypothesis that the auditory detection profile mirrors the irregularity trace. This is a tantalizing finding that nuances the way perceptual distortions affect action: a loss of perceptual resolution is reflected in a loss of playing stability in the absence of consistent timing deviations (irregularity). Finally, this result again undermines the interpretation that the instability peak at the octave boundary is related to a low-level motor process such as fingering. Such an interpretation would predict that there is no deterioration of auditory perception at the boundary, whereas our experiment shows there is.

Previous studies have investigated sensitivity to timing changes in regular sequences of events (Hyde and Peretz, 2004; Ehrlé and Samson, 2005). Typically, these paradigms employ sequences of five tones of which the fourth is delayed. Listening to deviations in two-octave scales is different since (i) the sequences are longer (15 notes), and (i) the items in the sequences are not identical, but vary with increasing pitch. Thus, the observed drop of temporal deviation detection at the octave boundary is likely the result of the auditory system’s dividing of the temporal sequence in the same, octave-unit chunks as was evident in the motor system. Indeed, previous studies using self-paced viewing of a slide show of actions showed longer looking times at boundaries of actions (Hard et al., 2011), suggesting increased processing demands at the boundaries of perceptual units. In our experiment, however, the notes arrived at fixed intervals beyond the control of participants. Therefore the increased processing load at the chunk boundaries is likely to have interfered with the processing of the items themselves, thus explaining our observed perceptual effect. As such, we predict that the same effect should be observable in the other scales (A-minor and F#-major) as well.

In sum, we have revealed a parallel between the instability trace in pianists’ playing and the detection rate of timing perturbations in listeners.

## General Discussion

### Overview

Our results indicate that it is possible to meaningfully dissociate irregularity as planned by the motor system and instability of the execution of the motor program. Experiment I revealed that these two factors contribute to the SD of the keystroke intervals as investigated in previous studies. The advantage of our analysis is that we tease apart systematic deviations, which can be rooted in perceptual biases (Penel and Drake, 1998) or residual expressive timing (Repp, 1999a), from mere motor execution errors. Another advantage is that the line fit can still be computed even if a few notes are missing or played twice. As a result, our analysis is robust enough to be applied to pianists who play relatively few correct scales, for example because of a movement disorder such as musician’s dystonia (Jabusch et al., 2004) or experimental design (Maidhof et al., 2009).

Note-by-note investigation reveals that instability is greater at the boundaries between the octaves. This is true for two-octave C-major scales and the motorically identical A-minor scales, revealing that it is not related to the C-major musical content but related to motor execution. We interpret these results as revealing that at the octave boundary a transition occurs between subsequent motor program chunks. Previous studies have interpreted the chunking as an aid to learning (Sakai et al., 2004), which is supported by evidence that in the course of learning, the smaller chunks are merged into larger ones (Rosenbaum et al., 1983). In our interpretation, this instability is a side-effect of loading the next motor sequence into the motor buffer (Lashley, 1951). Interestingly, our data stand in contrast to the previous findings of pianists’ tapping, showing reduced variability of taps at sequence boundaries (Loehr and Palmer, 2007). However, the latter study used much shorter sequences and calculate the variability of the intervals instead of note-by-note variability. These experimental differences may explain the different and robust findings in the present study.

The octave boundary instability is less strong but still present in the F#-major scales. This can readily be explained by the fact that F#-major scales are much less intensively practised because they are less common in the music literature. For example, one participant did not know it is played with a b rather than a b#. In other words, the F#-major scale may be represented more note-by-note in the motor system because it is played less frequently.

Indeed, we searched the ThemeFinder corpus (http://www.themefinder.org, containing 9792 themes at the time of our search). Of these, 936 themes were composed in C-major. Any theme in A-minor revealed 467 matches. F# major themes were only 139 in number. Indeed, these three counts are not independent: χ^2^(2) = 624.4, *p* < 0.001. In particular, there are between 2.8 and 3.2 (binomial 95% confidence interval) times more C-major themes than A-minor themes. Similarly, there are between 1.2 and 1.4 times (binomial 95% confidence interval) more A-minor than F#-major themes. Of course, some caution is needed in interpreting these corpus search results. They only concern themes and not entire pieces, which may contain modulations. Furthermore, the data is from classical and baroque periods only, and are therefore not necessarily the same as the typical pianists’ repertoire. However, it is likely that the distributions of tonalities are at least comparable. Furthermore, this difference between F#-major and C-major scales cannot be explained by washing out of the instability differences due to higher overall instability in the former case, because the mean instability is the same in both cases (Figure 3).

In order to further clarify the processing that occurs at the octave boundary, future investigation could add a weight to the wrist during scale playing, increasing its inertial mass. This means that the preparation for the thumb passage movement would have to be longer (Engel et al., 1997) and likely accompanied by more variability. Therefore, we predict the appearance of two additional peaks in the instability (at $\hat{4}$ and ${\hat{4}}^{\prime }$). However, great caution should be taken in such an endeavor, since the pianist’s muscular system is highly sensitive to changes in the playing environment (Sakai, 2002) and can easily result in injury. Alternatively, TMS could be applied during the production of the first octave, which would likely interrupt the output that is currently in the motor buffer. However, the second chunk (octave) is at that point not yet in the motor buffer and should therefore not be affected.

The picture that emerges from Experiment I is that the octave boundary instability is mainly a low-level motor sequencing phenomenon. But Experiment III reveals an unexpected parallel in perception: that the detection rate is lower at the boundaries of the two-octaves. Assuming that the two phenomena are causally related, which one is the cause and which the effect?

First we consider the possibility that the lack of auditory resolution at the octave boundary causes the playing to be less precise at those points. Similar hypotheses have been advanced that relate musical production to perception. One is that slowing down at the end is musically appropriate and not perceived as deviant (Repp, 1992b), and therefore played that way too. On another interpretation the perceptual space is non-veridical, with certain intervals (such as the last intervals of phrases) sounding shorter and therefore played longer: the perceptual hypothesis (Penel and Drake, 1998). In our case, the problem with both is that they cannot account for the detection impairment at the octave boundary, since we show that there is an increase in playing instability, but no systematic slowing (which would have appeared in the irregularity trace).

The perceptual hypothesis could be amended to include this possibility. Imagine that due to the lack of perceptual resolution at the octave boundary, the interval does not sound systematically shorter, but sometimes shorter and sometimes longer. As a result, the playing would sometimes compensate by playing it longer and sometimes by playing it shorter, yielding increased playing variability but not systematic deviation, in line with our findings. What is not satisfying about this explanation is that it does not account for why perceptual resolution is lower at such locations that do not seem musically meaningful. However, an even more immediate problem is that it would predict the octave boundary instability to be present equally in the F#-major scales, contrary to our findings.

A second explanation is the inverse causality: a lack in playing precision leads to impaired perception. Indeed, participants in Experiment III were musicians and could therefore be heavily influenced by exposure to musical material. A future study could decide this issue by performing the perception experiment on non-musicians. A limitation of such investigation will be that even non-musicians have much been passively exposed to music.

In sum, then, we conclude that the chunks formed in the motor system and in the perceptual system overlap, at least for the materials presently studied. How chunk formation in the two systems is causally related remains yet to be answered.

These questions open the road to further investigation into the relation between music perception and production, which may be more complex than previously accounted for. A future study may use a signal-detection-theoretical framework to tease apart response bias and sensitivity and correlate these to playing irregularity and inconsistency. Our prediction is that the irregularity trace will be mirrored by the detection bias, whereas the instability trace reflects the inverse of the sensitivity.

In sum, our study points to a dissociation between musical phrases and motor programs. Musical phrases have previously been found to be indicated by systematic slowing at the end (i.e., increased irregularity), whereas our finding is that motor sequences are demarcated by increased playing instability. Perhaps the two reflect the previously discovered dissociation between timing processes and item sequencing (Pfordresher, 2003). This issue could be further clarified by testing whether increased keystroke variability is found at the boundary of learned sequences in a serial reaction time paradigm (Nissen and Bullemer, 1987), as our interpretation would predict.

One limitation in the current study is that although contrary to previous studies we have included scales of different tonalities, still all our material consisted of two-octave scales. A future study could investigate scales over three octaves, although caution would need to be taken to control for the larger distance the arm needs to cover to reach the three octaves.

## Conflict of Interest Statement

The authors declare that the research was conducted in the absence of any commercial or financial relationships that could be construed as a potential conflict of interest.
